# A complex selection signature at the human *AVPR1B *gene

**DOI:** 10.1186/1471-2148-9-123

**Published:** 2009-06-01

**Authors:** Rachele Cagliani, Matteo Fumagalli, Uberto Pozzoli, Stefania Riva, Matteo Cereda, Giacomo P Comi, Linda Pattini, Nereo Bresolin, Manuela Sironi

**Affiliations:** 1 Scientific Institute IRCCS E. Medea, Bioinformatic Lab, Via don L. Monza 20, 23842 Bosisio Parini (LC), Italy; 2 Bioengineering Department, Politecnico di Milano, P.zza L. da Vinci, 32, 20133 Milan, Italy; 3 Dino Ferrari Centre, Department of Neurological Sciences, University of Milan, IRCCS Ospedale Maggiore Policlinico, Mangiagalli and Regina Elena Foundation, Via F. Sforza 35, 20100 Milan, Italy

## Abstract

**Background:**

The vasopressin receptor type 1b (*AVPR1B*) is mainly expressed by pituitary corticotropes and it mediates the stimulatory effects of AVP on ACTH release; common *AVPR1B *haplotypes have been involved in mood and anxiety disorders in humans, while rodents lacking a functional receptor gene display behavioral defects and altered stress responses.

**Results:**

Here we have analyzed the two exons of the gene and the data we present suggest that *AVPR1B *has been subjected to natural selection in humans. In particular, analysis of exon 2 strongly suggests the action of balancing selection in African populations and Europeans: the region displays high nucleotide diversity, an excess of intermediate-frequency alleles, a higher level of within-species diversity compared to interspecific divergence and a genealogy with common haplotypes separated by deep branches. This relatively unambiguous situation coexists with unusual features across exon 1, raising the possibility that a nonsynonymous variant (Gly191Arg) in this region has been subjected to directional selection.

**Conclusion:**

Although the underlying selective pressure(s) remains to be identified, we consider this to be among the first documented examples of a gene involved in mood disorders and subjected to natural selection in humans; this observation might add support to the long-debated idea that depression/low mood might have played an adaptive role during human evolution.

## Background

The neurohypophyseal peptide vasopressin (AVP) is involved in different physiological functions, including stimulation of liver glycogenolysis, contraction of vascular smooth muscle cells, antidiuresis and platelet aggregation (reviewed in [1]). In addition, AVP plays an important role as a regulator of the hypothalamic-pituitary-adrenal (HPA) axis [2,3]. AVP receptors are G protein-coupled and can be divided in three subtypes: V1a, V1b, and V2, encoded in humans by *AVPR1A*, *AVPR1B *and *AVPR2*, respectively (reviewed in [1]). The V2 receptor is primarily expressed in the kidney and it controls renal collecting duct water permeability. *AVPR1A *has wider expression and it regulates physiological effects such as vascular cell contraction, glycogenolysis and platelet aggregation. The type 1b receptor is mainly expressed by pituitary corticotropes and it mediates the stimulatory effects of AVP on ACTH release. Nonetheless, *AVPR1B *expression has also been described in many brain areas [4,5] and in different peripheral tissues [4], while recent evidences have indicated that AVP can induce glucagone and insulin secretion from isolated rodent pancreatic islets through the V1b receptor [6,7].

Recently, considerable attention has been placed on the role of AVP and its receptors in complex behavioral tracts. Indeed, variations the *AVPR1A *promoter region have been shown to influence reproductive and social behavior in voles [8], as well as complex behavioral traits in humans such as altruism [9], reproductive attitudes [10,11] and creative dance performance [12]. Therefore, different studies [8,13] have analyzed the evolutionary history of the type 1a receptor in different mammalian species. In comparison, *AVPR1B *has attracted less attention, although data from knock-out mice (*V1bR*^-/-^) indicate that it plays central roles in both behavioral and metabolic systems. Its regulatory function on the HPA axis is demonstrated by the reduced levels of circulating ACTH and corticosterone under both stress and resting conditions in *V1bR*^-/- ^animals [2]. These mice also exhibit limited aggressive behavior [14] and reduced ultrasonic vocalizations in different social contexts [15]. Interestingly, a selective V1b antagonist produces anxiolytic- and antidepressant-like effects in rodents [16] and in humans *AVPR1B *variants have been associated with recurrent major depression [17], early-onset mood disorders [18] and panic disorder [19]. In line with these findings, the receptor has been proposed as a possible therapeutic target in stress-related disorders [20].

## Methods

### DNA samples and sequencing

Human genomic DNA was obtained from the Coriell Institute for Medical Research. The genomic DNA of one gorilla and one gibbon was derived from the European Collection of Cell Cultures (ECACC). All analyzed regions were PCR amplified and directly sequenced; primer sequences are available upon request. PCR products were treated with ExoSAP-IT (USB Corporation Cleveland Ohio, USA), directly sequenced on both strands with a Big Dye Terminator sequencing Kit (v3.1 Applied Biosystem) and run on an Applied Biosystems ABI 3130 XL Genetic Analyzer (Applied Biosystem). Sequences were assembled using AutoAssembler version 1.4.0 (Applied Biosystems), and inspected manually by two distinct operators.

### Data retrieval and haplotype construction

Genotype data for Yoruba (YRI) and Europeans (EU) were retrieved from the SeattleSNPs website [21]. Genotype data for 238 resequenced human genes were derived from the NIEHS SNPs Program web site [22]. We selected genes that had been resequenced in populations of defined ethnicity including African American (AA), EU, YRI and East Asians (AS) (NIEHS panel 2). In particular, for each NIEHS gene a 2 kb region was randomly selected; the only requirement was that it did not contain any resequencing gap. Haplotypes were inferred using PHASE version 2.1 [23,24], a program for reconstructing haplotypes from unrelated genotype data through a Bayesian statistical method. Haplotypes for individuals resequenced in this study are available as supplemental material (Additional File 1).

### Statistical analysis

Phylogenetic relationships among primate *AVPR1B *genes were reconstructed by obtaining a tree with use of MrBayes [25]. In particular, we run a Markov chain for 1 million cycles under the HKY85 model of DNA substitution with no rate variation across sites.

The ratio of d_N _over d_S _was calculated using CODEML in the PAML Package (v.3.15) [26]. We used the so-called "free-ratio" model in which dn/ds is free to vary among branches with no variation among sites.

Linkage disequilibrium analyzes were performed using the Haploview software (v. 4.1) [27] and blocks were identified through the implemented confidence interval algorithm [28]. In particular, marker pairs are defined to be in "strong LD" if the one-sided upper 95% confidence bound on D' is >0.98 and the lower bound is above 0.7; a block is created when if 95% of informative comparisons are in "strong LD".

Tajima's D [29], Fu and Li's D* and F* [30] statistics, as well as diversity parameters θ_W _[31] and π [32] were calculated using *libsequence *[33], a C++ class library providing an object-oriented framework for the analysis of molecular population genetic data. Coalescent simulations were performed using the *cosi *package [34] and its best-fit parameters for YRI, AA, EU and AS populations with 10000 iterations. Additional coalescent simulations were computed with the *ms *software [35] specifying the number of chromosomes, the mutation parameter estimated from the data, and the recombination rate with 10000 iterations for each demographic model. The other parameters for each model were set as previously proposed [36,37]. Estimates of the population recombination rate parameter ρ were obtained with the use of the Web application MAXDIP [38].

The Maximum-likelihood-ratio HKA test was performed using the MLHKA software [39] using multi-locus data of 15 NIEHS genes (reference genes) and *Pan troglodytes *(NCBI panTro2) as an outgroup. The 15 reference genes were randomly selected among NIEHS loci shorter than 20 kb that have been resequenced in the 4 populations (panel 2). The reference set was accounted for by the following genes: *ENO1, VNN2, MMP12, GLRX, CHRNA4, SULT1C2, PRDX6, H2AFX, ODC1, MT2A, RETN, CYP4B1, RECQL4, MCL1 *and *MB*. In particular, we evaluated the likelihood of the model under two different assumptions: that all loci evolved neutrally and that only the region under analysis was subjected to natural selection; statistical significance was assessed by a likelihood ratio test. We used a chain length (the number of cycles of the Markov chain) of 2 × 10^5 ^and, as suggested by the authors [39], we ran the program several times with different seeds to ensure stability of results. A second multi-locus HKA test was performed using the "HKA" software distributed by Jody Hey [40] and the same reference loci reported above; 1000 coalescent simulations were performed with the *cosi *package [34]. In both cases only neutrally evolving sites were considered.

Median-joining networks to infer haplotype genealogy was constructed using NETWORK 4.5 [41]. Estimate of the time to the most common ancestor (TMRCA) was obtained using a phylogeny based approach implemented in NETWORK 4.5 using a mutation rate based on the number of fixed differences between chimpanzee and humans. A second TMRCA estimate derived from application of a maximum-likelihood coalescent method implemented in GENETREE [42,43]. Again, the mutation rate μ was obtained on the basis of the divergence between human and chimpanzee and under the assumption both that the species separation occurred 6 MYA and of a generation time of 25 years. Using this μ and θ maximum likelihood (θ_ML_), we estimated the effective population size parameter (N_e_). With these assumptions, the coalescence time, scaled in 2N_e _units, was converted into years. For the coalescence process, 10^6 ^simulations were performed. A third TMRCA was calculated as previously proposed [44] that calculates the average nucleotide diversity between the MRCA and each of the chromosomes.

All calculations were performed in the R environment [45].

### Molecular modeling

The three-dimensional model of the human V1b receptor (Swiss-Prot entry: P47901) was obtained by comparative modelling using the known crystal structure of the closely related bovine rhodopsin (Protein Data Bank ID 1u19) as a template, as provided by MODBASE [46]. The significance of the alignment between target and template sequences is E = 4e^-87^. The retrieved structure was rendered with FirstGlance in Jmol [47].

## Results

### *AVPR1B *evolution in primates

**Figure 1:**
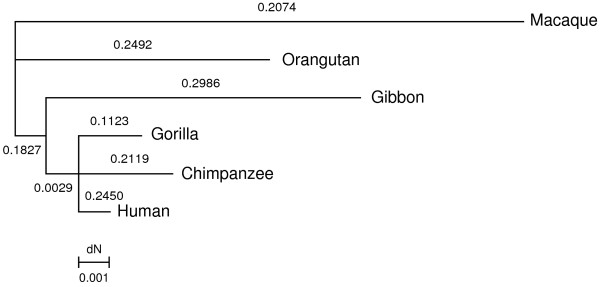
**Phylogenetic tree of *AVPR1B *coding region**. The d_N_/d_S _ratio (ω) is reported for each branch. Branch lengths correspond to d_N_.

As a first step, we wished to gain insight into the evolutionary history of *AVPR1B *in primates; to this aim the two exons (including the 5' and 3'UTRs) were sequenced from gorilla and gibbon genomic DNA, while the gene sequences for additional primate species (namely, chimpanzee, orangutan and macaque) were retrieved from public databases. A phylogenetic tree (Fig. 1) was produced for the 6 primates with the use of MrBayes [25]. It is worth noting that the failure to resolve the human-chimpanzee-gorilla trichotomy is likely due to the short span of the analyzed region (a total of ~1.7 kb). Using the free ratio model we calculated the d_N_/d_S _ratio (ω) along all lineages; in all cases low ratios are observed indicating that *AVPR1B *has evolved under purifying selection in primates. Calculation of ω for the human-chimpanzee pairwise alignment resulted in a value of 0.28, comparable to the average value for all human genes (ω = 0.23) [48].

### Nucleotide diversity and neutrality tests

We next aimed at analyzing the evolution of *AVPR1B *in human populations. We therefore resequenced the two exons of *AVPR1B *and their flanking sequences (including the putative promoter, part of the intron and the 3'UTR) in two populations of Asian and African American ancestry. Additional data referring to YRI and EU subjects were derived from the SeattleSNPs website. The final data set was accounted for by 96 individuals belonging to 4 ethnically distinct populations. A total of 37 SNPs were identified; among these 3 and 1 nonsynonymous substitutions were located in exon 1 (Lys65Asn, Gly191Arg and Ser267Gly) and exon 2 (Arg364His), respectively. Three synonymous coding variants were also identified (Leu130Leu and His224His in exon 1 and Ser373Ser in exon 2).

Analysis of linkage disequilibrium indicated that *AVPR1B *lies on a single haplotype block in EU and AS, but not in African populations (see Additional File 2). Nucleotide diversity was assessed using two indexes: θ_W _[31], an estimate of the expected per site heterozygosity, and π [32] the average number of pairwise sequence nucleotide differences. In order to compare the values we obtained for the two *AVPR1B *exons, we calculated θ_W _and π for a set of randomly selected 2 kb windows deriving from 238 genes resequenced by the NIEHS program in the same population samples; the percentile rank corresponding to exon 1 (Ex1) and 2 (Ex2) in the distribution of values for reference 2 kb windows is reported in table 1 and indicates that Ex2 displays high nucleotide diversity in all populations, despite showing a level of human-chimpanzee divergence (0.011) comparable to the genome average [49]; the same holds true for Ex1 when African subjects are considered, while AS and EU show no unusual nucleotide variation pattern in this gene region. Under neutral evolution, values of θ_W _and π are expected to be roughly equal; for both Ex1 and Ex2 this is not verified in most cases (Tab. 1) and we therefore wished to investigate whether *AVPR1B *might be subjected to natural selection in humans. Widely used neutrality tests include Tajima's D (D_T _[29]) and Fu and Li's D* and F* [30]. D_T _evaluates the departure from neutrality by comparing θ_W _and π. Positive values of D_T _indicate an excess of intermediate frequency variants and are an hallmark of balancing selection; negative D_T _values indicate either purifying selection or a high representation of rare variants as a result of a selective sweep. Fu and Li's F* and D* are also based on SNP frequency spectra and differ from D_T _in that they also take into account whether mutations occur in external or internal branches of a genealogy. Since, population history, in addition to selective processes, is known to affect frequency spectra and all related statistics, we performed coalescent simulations for all populations using a model that incorporates demographic scenarios [34]. Additional demographic models [36,37] were used for coalescent simulations and the results, which confirm those reported below, are available as additional file 3. Also, in order to disentangle the effects of selection and population history, we exploited the fact that selection acts on a single locus while demography affects the whole genome: as a control data set we therefore calculated test statistics for the 2 kb reference windows deriving from NIEHS genes. Neutrality tests for Ex1 were consistent with neutrality for African populations, while marginally significant negative values of Fu and Li's F* and D* where obtained for EU. In the case of AS only 3 segregating SNPs are observed in the region.

**Table 1:** Summary statistics for *AVPR1B*.

**Region**	**Pop.^a^**	**N^b^**	**S^c^**	**π^d^**		**θ**_**W**_^**e**^		**Tajima's D^f^**		Fu and Li's D*^f^		Fu and Li's F*^f^	
								
					rank^g^		rank^g^		rank^g^		rank^g^		rank^g^
Exon 1 (2268 bp)	**YRI**	48	15	19.22	0.96	14.90	0.85	0.89 (0.046)	0.90	0.21 (0.25)	0.67	0.52 (0.13)	0.76
	**AA**	48	13	12.78	0.84	12.91	0.77	-0.03 (0.26)	0.68	-1.52 (0.15)	0.15	-1.20 (0.25)	0.25
	**EU**	46	8	4.71	0.50	8.02	0.73	-1.13 (0.22)	0.16	-2.20 (0.048)	0.05	-2.18 (0.042)	0.05
	**AS**	50	3	1.90	0.28	2.95	0.30	-0.69 (0.22)	0.32	0.89 (0.17)	0.83	0.48 (0.33)	0.68
													
Exon 2 (2112 bp)	**YRI**	48	20	32.67	0.99	19.08	0.94	1.71 (0.005)	0.97	1.32 (0.019)	0.96	1.72 (0.002)	0.99
	**AA**	48	19	32.01	>0.99	20.26	0.96	1.84 (0.003)	0.99	1.66 (<0.001)	>0.99	2.04 (<0.001)	>0.99
	**EU**	46	14	22.53	0.98	15.05	0.96	1.52 (0.062)	0.90	1.54 (0.002)	>0.99	1.81 (0.009)	0.98
	**AS**	50	14	12.17	0.88	14.77	0.97	-0.54 (0.275)	0.39	1.54 (0.002)	>0.99	0.99 (0.177)	0.86

^a ^population;

^b ^sample size;

^c ^number of segregating sites;

^d ^θ_w _estimation per site (× 10^-4^);

^e ^π estimation per site (× 10^-4^);

^f ^*p *values in parenthesis are obtained by applying a population genetics model that incorporated demographic models [34];

^g ^percentile rank relative to the distribution of 2 kb reference windows genes.

In contrast, data for Ex2 indicate departure from neutrality in most populations (excluding AS) with significantly positive values for most statistics. In line with these findings, D_T_, as well as Fu and Li's F* and D* calculated for *AVPR1B *Ex2 rank above the 95^th ^percentile of the distribution of reference 2 kb windows in non-Asian populations. These latter results suggest that nucleotide diversity in Ex2 has been shaped by balancing selection; conversely, the negative statistics observed for EU at Ex1 can in principle be explained by either purifying selection or directional selection since both processes result in an excess of low frequency variants. Fay and Wu's H [50] is usually applied to distinguish between the two possibilities. Negative H values indicate an excess of high frequency derived alleles, a finding consistent with the action of directional but not purifying selection. Calculation of Fay and Wu's H for EU resulted in a significantly negative value (H = -9.27, *p *= 0.0006).

A striking feature of these results is the large difference in D_T _between Ex1 and Ex2 we observe in the EU sample. Such a marked variation in the allele frequency spectrum is even more impressive in light of the strong linkage disequilibrium between the two exons in Europeans (see Additional File 2). In order to evaluate whether such change in the frequency spectrum might be due to chance alone, we performed 10,000 coalescent simulations by generating gene genealogies for a 8.5 kb region (corresponding to the *AVPR1B *gene); simulations were performed with the estimated recombination rate for *AVPR1B *and using the *cosi *package with its best-fit parameters for EU [34]. The simulated samples were then treated as the gene and D_T _was calculated for the first 2268 bp and the last 2112 bp (corresponding to Ex1 and Ex2). The results indicated the probability of observing a difference in D_T _values as large as or larger than that we observe for the two *AVPR1B *exons amounts to 0.035, therefore rejecting a neutral scenario.

Under neutral evolution, the amount of within-species diversity is predicted to correlate with levels of between-species divergence, since both depend on the neutral mutation rate [51]. The HKA test [52] is commonly used to verify whether this expectation is verified. Here we applied a Maximum-Likelihood-ratio HKA (MLHKA) test [39] by comparing polymorphisms and divergence levels at *AVPR1B *Ex1 and Ex2 with 15 NIEHS genes resequenced in the four populations we analyzed (see methods). The results are shown in table 2 and indicate that for Ex1 a reduction in nucleotide diversity versus divergence is detectable in the AS sample although the result is not statistically significant. The opposite situation is verified at Ex2, a significant excess of polymorphisms being observed in all populations. The MLHKA test is relatively robust to demography given its multi-locus comparison framework [39]; still, while this method is conservative in cases of population expansion (i.e. for populations of African origin), population size bottlenecks might artificially result in significant p values [39]. In order to evaluate whether this is the case, a second multi-locus HKA test was performed using the "HKA" software [40] which allows estimation of statistical significance through coalescent simulations. These latter were performed using a previously describe demographic model [34] as above and significant results were obtained for both EU and AS (Tab. 2); in both cases the test of maximum cell value [53] indicated *AVPR1B *Ex2 as an outlier (p = 0.018 and 0.001 for EU and AS, respectively).

**Table 2:** Multi-locus HKA test results for *AVPR1B *exons.

**Region**	**Fixed sub.**	**MLHKA**								**Multi-locus HKA**			
			
		**YRI**		**AA**		**EU**		**AS**		**EU**		**AS**	
							
		k^a^	*p*^b^	k^a^	*p*^b^	k^a^	*p*^b^	k^a^	*p*^b^	L^c^	*p*^d^	L^c^	*p*^d^
Ex1	35	2.08	.086	2.06	.20	1.60	.54	0.66	.13	n.a.^e^	n.a.^e^	n.a.^e^	n.a.^e^
Ex2	25	3.77	.0046	3.38	.0093	3.56	.011	3.50	.011	35	.001	44	<.001

^a ^k (selection parameter) values from MLHKA test; k<1 means a reduction in polymorphism while k>1 means an excess of nucleotide variation;

^b ^*p *values for the MLHKA test;

^c ^likelihood (sum of deviations) for multi-locus HKA test;

^d ^*p *values from demographic coalescent simulations for multi-locus HKA;

^e ^not analyzed.

**Figure 2:**
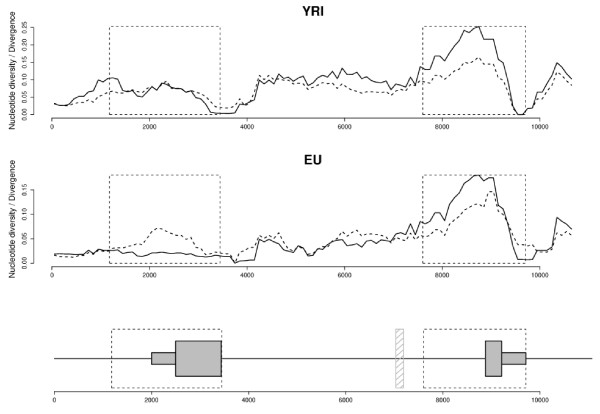
**Sliding window analysis of θ_W _and π over human-chimpanzee divergence along the *AVPR1B *gene region**. Plots were separately performed for YRI (upper panel) and EU (lower panel). The intron-exon gene structure is also shown. Both θ_W _(hatched line) and π (full line) are normalized to the human-chimpanzee divergence; windows of 1000 bp with a step of 100 bp were used. The shaded box indicates a resequencing gaps; the boxed regions are those we resequenced and analyzed.

The signatures of balancing selection are predicted to extend over short genomic distances [54,55]; nonetheless we wished to verify that the evolution of Ex2 is not influenced by the presence of a linked balanced polymorphism located elsewhere. To this aim, we exploited the availability in SeattleSNPs of resequencing data covering the whole gene and extending 2 kb downstream in the intergenic region. We calculated the ratio of both θ_W _and π to human-chimpanzee divergence in sliding windows for YRI and EU. As shown in figure 2, a peak in both ratios is observed at Ex2 in both populations. This peak decays in the flanking intergenic region.

**Figure 3:**
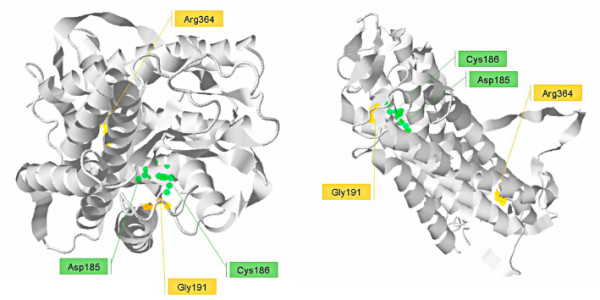
**Schematic representation of the three-dimensional model of the V1b receptor**. Two different orientations of the modeled protein are shown. The 191 and the 364 residues are highlighted (orange halos). The former is in close proximity to the Asp185 and Cys186 residues (green halos) that are known to be in close contact with AVP.

As far as coding variants are concerned, analysis of SNPs in Ex1 indicated that the derived Gly191 allele has risen to high frequency in most populations and is fixed among AS. With respect to exon 2, a replacement variant (His364 allele) displays higher frequency in EU and AS compared to African populations. Similarity to the other AVP receptors, *AVPR1B *belongs to the 7-transmembrane receptor family; residue 191 is located in the second extracellular loop of the receptor, a region which is believed to be important for the binding properties of these molecules [56], including *AVPR1A *[57]. Indeed, modeling of the protein (Fig. 3) indicated that the 191 residue is located in close proximity to Asp185 and Cys186, two residues in close contact with AVP [58]. With respect to residue 364, it is located in the intracellular C-terminal tail, a protein region involved in molecular interactions with adaptors and scaffolding proteins [59].

### Haplotype analysis

**Figure 4:**
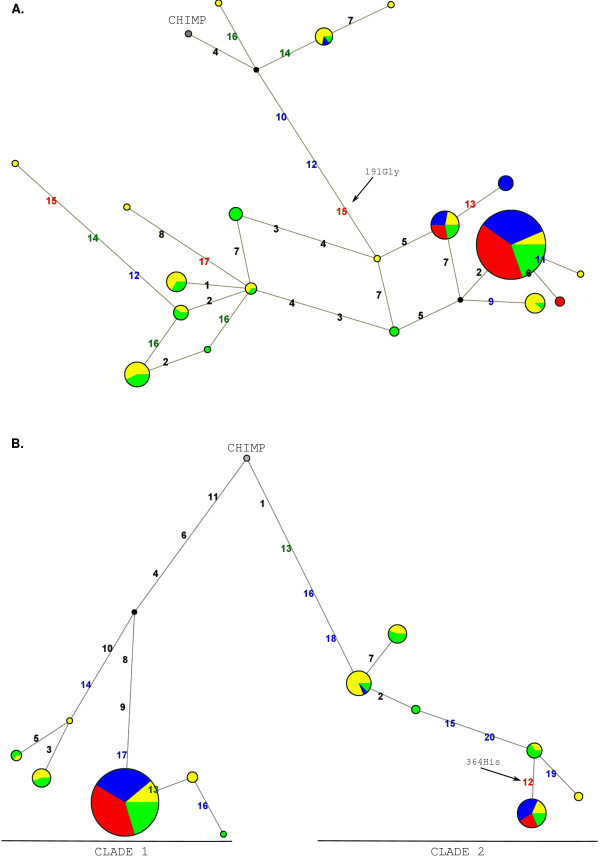
**Genealogy of A *VPR1B *Ex1 (A) and Ex2 (B) haplotypes reconstructed through median-joining networks**. Each node represents a different haplotype, with the size of the circle proportional to the haplotype frequency. Also, circles are color-coded according to population (yellow: AA, blue: EU, green: YRI, red: AS). Polymorphisms are shown in red, green, blue or black if they are nonsynonymous, synonymous, located in UTRs or introns, respectively.

In order to study the genealogy of Ex1 and Ex2 haplotypes we constructed median-joining networks [41]. The topology of Ex1 genealogy indicates that, while African chromosomes are relatively diverged from one another, most Asian and European haplotypes are clustered and all carry the Gly191 allele (Fig. 4A).

With respect to Ex2 genealogy, two major clades separated by long branch lengths are evident (Fig. 4B), each containing common haplotypes. Some population differentiation is also present along clade 2 since most non-African chromosomes are identical and carry the His364 allele, while YRI and AA also display common haplotypes carrying the Arg364 allele.

**Figure 5:**
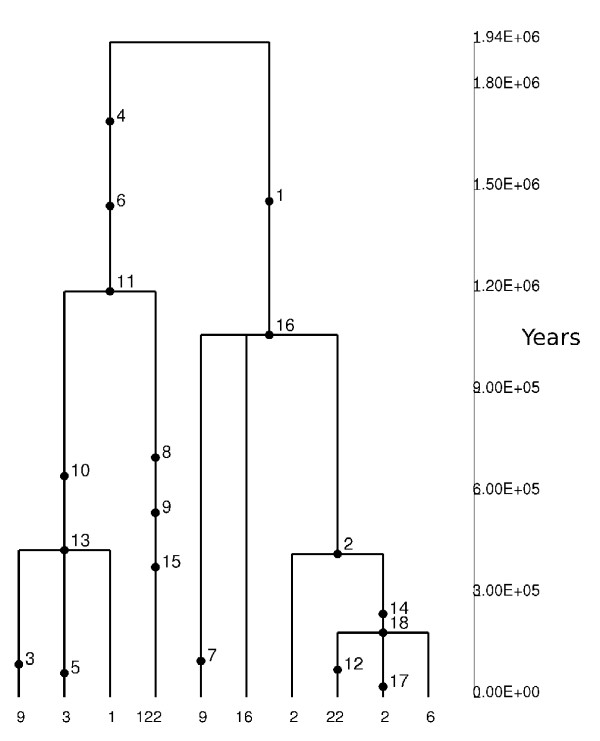
**Estimated tree for the *AVPR1B *Ex2 gene region**. Mutations are represented as black dots and named for their physical position along Ex2 region. Mutation numbering does not correspond to the one shown in figure 4B since, as reported in the text, 2 SNPs were removed as they violated the infinite site model. The absolute frequency of each haplotype is also reported.

In order to estimate the TMRCA (Time to the Most Recent Common Ancestor) of the two Ex2 haplotype clades, we applied a phylogeny-based method [41] based on the measure ρ, the average pairwise difference between the two haplotype clusters. ρ resulted equal to 6.05, so that using a mutation rate based on 35 fixed differences between chimpanzee and humans and a separation time of 6 million years (MY) [60], we estimated a TMRCA of 3.19 MY years (SD: 673 KY). Given the low recombination rate in the region, we wished to verify this result using GENETREE, which is based on a maximum-likelihood coalescent analysis [42,43]. The method assumes an infinite-site model without recombination and, therefore, haplotypes and sites that violate these assumptions need to be removed: in this case, only 2 single segregating sites had to be removed. The resulting gene tree, rooted using the chimpanzee sequence, is partitioned into two deep branches (Fig. 5). A maximum-likelihood estimate of θ (θ_ML_) of 2.5 was obtained, resulting in an estimated effective population size (N_e_) of 13043, a value comparable to most figures reported in the literature [61]. Using this method, the TMRCA of the Ex2 haplotype lineages amounted to 1.94 MY (SD: 497 KY). A third TMRCA of 3.67 MY was estimated by applying a previously described method [44] that calculates the average sequence divergence separating the MRCA and each of the chromosomes; coalescence time is then obtained by scaling this average divergence to the mutation rate obtained from human-chimpanzee divergence in the region.

All TMRCA estimates indicate an unusually deep coalescent time, as estimates for neutrally evolving autosomal loci range between 0.8 and 1.5 MY [61]. Deep haplotype genealogies might result from both balancing selection and ancient population structure (reviewed in [62]). Yet, balancing selection is expected to elongate the entire neutral genealogy, while the effects of ancient population structure are reflected in an increase in the genealogical time occupied by single lineages [63,64]. A possibility to discriminate between these two scenarios is to calculate the percentage of congruent mutations, meaning those that occur on the basal branches of a genealogy [64]. When we applied this approach to the Ex2 genealogy, a percentage of congruent mutations equal to 25% was obtained; this is much lower than previous estimates under a model of ancient population structure, which ranged from 42 to 45% [65,66], indicating that balancing selection rather than population subdivision is responsible for the maintenance of the two clades.

## Discussion

The interest in the identification and analysis of genomic regions subjected to non-neutral evolution is at least twofold. First, such studies provide insight into the evolutionary history of our species, with special reference to the adaptive events underlying phenotypic changes between humans and primates/hominids. Second, these analysis are expected to result in the identification of functional variants, which in turn, might influence disease susceptibility or drug response. Analysis of *AVPR1B *evolutionary history in humans might fit both these aims since the gene has been involved in complex behavioral traits in other mammals [14-16] and it has been associated with psychiatric diseases [17-19]. Also, the recently proposed idea [20] of the receptor as a potential therapeutic target in stress-related disorders suggests that the identification of functional variants will be valuable in the field of pharmacogenetics.

Here we have analyzed the two exons of the gene and, overall, the data we present suggest that *AVPR1B *has been subjected to natural selection. Analysis of Ex2 strongly suggests the action of balancing selection in African populations and Europeans: the region displays high nucleotide diversity, an excess of intermediate-frequency alleles, a higher level of within-species diversity compared to interspecific divergence and a genealogy with common haplotypes separated by deep branches. Yet, this relatively unambiguous situation is complicated by the presence of unusual features across Ex1. The possibility that a variant in this region has been subjected to directional selection in EU and has reached fixation in AS is supported by few observations: the large variance of D_T _in EU and the significantly negative H value in this same population. Yet, population structure and different demographic scenarios can yield statistically significant H values, as well [67]. Also, few theoretical models are available to evaluate the difference in D_T _at different loci; by performing coalescent simulations we verified that such a shift in the allele frequency spectrum is unlikely to be due to chance; consistently, previous descriptions [68,69] of marked D_T _differences at linked regions were ascribed to natural selection. Yet, although the closest upstream gene is located more than 72 kb apart, we cannot exclude that variants in Ex1 are hitchhiking with a distant selected allele. Also, the possibility that non-selective forces are responsible for this finding cannot be ruled out and the features we observe might be due to balancing selection acting on exon 2, with Ex1 being neutrally evolving. Although we cannot provide definitive evidence for directional selection at Ex1, it is worth mentioning that the Gly191Arg variant might be a good candidate to represent a functional SNP: as mentioned above, it is located in the second extracellular loop, in close proximity to residues involved in AVP binding (Fig. 3); support to the possible functional significance of this aminoacid replacement, comes from the observation that mutagenesis of most residues in the second extracellular loop of *AVPR1A *results in altered AVP binding properties [57]; also different missense substitutions in the same region of *AVPR2 *have been described to be pathogenetic and cause nephrogenic diabetes insipidus [70-72]. With respect to the possible selection targets in Ex2, SNPs located in the 3'UTR (figure 4B) represent possible candidates, although the splitting of clade 2 into two haplotype clusters with geographically differentiated distribution suggests that additional variants (either the Arg364His SNP or other UTR polymorphisms) might have been subjected to some selective pressure, as well. Of course, specific experiments will be needed to verify whether this prediction is verified. Similarly, a source of selective pressure must be envisaged to explain our findings. Unfortunately not much is known on the function and regulation of *AVPR1B *in humans; moreover, the gene is not covered by any HapMap SNP at the moment, so that it is likely to be excluded by most genome-wide association studies. Instead, some information about the gene derives from experiments in rodents where *AVPR1B *function is abolished by either genetic manipulation or antagonist administration; these experiments imply the inherent difficulty in translating mouse phenotypes to humans, which is even more complicated in the case of genes involved in complex behavioral traits, as *AVPR1B *is suggested to be. Given this premise, different possibilities can be envisaged for selection to act on *AVPR1B*, including its role in response to stress, psychological/behavioral manifestations and metabolic control. This latter aspect mainly refers to the expression of *AVPR1B *in human pancreas; experiments on isolated human islets cells, as well as in rodents, indicate that AVP can induce both glucagon and insulin secretion via binding to *AVPR1B *[6,73,74]. Also, *V1bR*^-/-^mice display insulin hypersensitivity [73] possibly due to altered signaling in adipocytes. The possibility therefore exists that one or more variants in *AVPR1B *have played an adaptive role as thrifty alleles [75] by favoring energy storage in times of low food availability. Indeed, long-standing balancing selection at the *CAPN10 *locus, also involved in insulin secretion and action [76], has previously been demonstrated [77]. In both cases long-term fluctuations in environmental conditions and food availability might be regarded as an explanation for the maintenance of balanced polymorphisms. Nonetheless, our knowledge on the regulation of *AVPR1B *signaling in peripheral tissues is too limited to allow extensive speculation on this issue. Conversely, the role of AVP in the stress response is better characterized; in response to stressful stimuli, AVP acts synergistically with corticotrophin-releasing hormone (CRH) to facilitate ACTH release, resulting in a consequent increase in plasma corticosterone/cortisol concentration (reviewed in [78]). Such AVP effect is mediated by *AVPR1B *[1] and ACTH measurements in mice have indicated that AVP plays important roles in response to some acute stressors (e.g. hypoglycemia, forced swim stress, lipopolysaccharyde challenge and ethanol intoxication, change in environment), as well as in times of chronic stress [2,79-83]. Stress responses have an evident adaptive significance and involve both physical and behavioral strategies, with the signaling pathway of *AVPR1B *being possibly involved in both. Besides the reported associations between *AVPR1B *and mood or anxiety disorders [17-19], evidences that the receptor has a role in behavioral traits come from the observation that blocking its activity in mice (either by gene knock-out or pharmacologically) results in decreased aggressive behaviors [14], reduced social interactions [15] and anxiolytic- and antidepressant-like effects [16]. In both humans and animals (reviewed in [84]), behavioral responses can be extremely different among individuals of the same species (reviewed in [84,85]) and recent observations (reviewed in [84,85]) indicate the maintenance of different behavioral strategies within populations to be adaptive. Frequency-dependent selection is deemed responsible for the coexistence of behavioral types in populations [86] and, in general, social interaction behaviors are thought to have frequency-dependent payoffs [86-88]. Frequency-dependent selection is among the possible causes for the maintenance of balanced alleles; it is therefore conceivable that a portion of genes involved in behavioral traits have evolved under a balancing selection regime, *AVPR1B *possibly representing one such example. An alternative and non mutually-exclusive explanation for the maintenance of variability in behavior-related traits is response to changing environmental conditions (reviewed [85]), which is also expected to result in balancing selection regimes. Clearly, different behavioral strategies can be advantageous or disadvantageous depending on the environment; for example, in unpropitious situations organisms might benefit from down-regulating effort and risk taking, the opposite being true when the environment is more favorable. These explanations have been regarded as possibly supporting an adaptive origin for depression/low mood in humans [89], a condition which has been associated to polymorphisms in *AVPR1B *[17,18]. The evolutionary hypothesis for depression has been highly debated in recent scientific literature (see [89] for a review) and is based both on the high incidence of this condition in modern populations [89] and on different observations. For example, anxiety can be advantageous in perceiving danger [90], a depressive state correlates positively with harm avoidance [91] and favors disengagement from unproductive efforts [89]. Obviously, these attitudes are selectively advantageous in some environmental conditions (i.e. when action is futile or dangerous) but not in others. Following this line, balancing selection rather than directional or positive selection might be expected at loci which regulate such behavioral traits.

## Conclusion

Up to now the evolutionary theory of depression has received few demonstrations. To our knowledge, the one we report here might be the first documented example of a gene involved in mood disorders and subjected to natural selection during human evolutionary history. Nonetheless, it is worth mentioning that the two reports associating *AVPR1B *variants to depression/mood disorders identified the same major haplotype as being either protective or predisposing in different population of European descent. Dempster et al. [18] suggested that the discrepancy might result from the use of slightly different sets of markers; we analyzed the haplotype structure of the entire gene in EU (not shown) and found little support for this possibility. Therefore, further studies will be required before a full knowledge can be obtained of *AVPR1B *evolutionary role as well as of its association with mental diseases.

## Abbreviations

AVP: arginin vasopressin; HPA: hypothalamic-pituitary-adrenal; CRH: corticotrophin-releasing hormone; ACTH: adrenocorticotropic hormone; AA: African American; EU: Europeans; YRI: Yorubans; AS: East Asians; TMRCA: time to the most common ancestor; MY: million years.

## Authors' contributions

RC and SR performed all resequencing experiments and analyzed the data; MF performed population genetics analyzes; MS, MF, RC, GPC and UP analyzed and interpreted the data; NB participated in the study coordination; LP performed protein structure analyzes; MC analyzed *AVPR1B *evolution in primates. MS conceived the study and wrote the paper.

## Supplementary Material

Additional file 1 **Haplotypes for individuals resequenced in this study.** Click here for file

Additional file 2 **Linkage disequilibrium analysis for the genomic region encompassing *AVPR1B *exons 1 and 2.** Click here for file

Additional file 3 **Summary statistic p values obtained using different demographic models.** Click here for file
