# Supplementary material for: A complex selection signature at the human AVPR1B gene
Source: BMC Evol Biol. 2009 Jun 1;9:123. doi: 10.1186/1471-2148-9-123 (PMC2700802; doi:10.1186/1471-2148-9-123)
Supplement: Additional file 2 — Linkage disequilibrium analysis for the genomic region encompassing AVPR1B exons 1 and 2. [file 1471-2148-9-123-S2.pdf]

A.

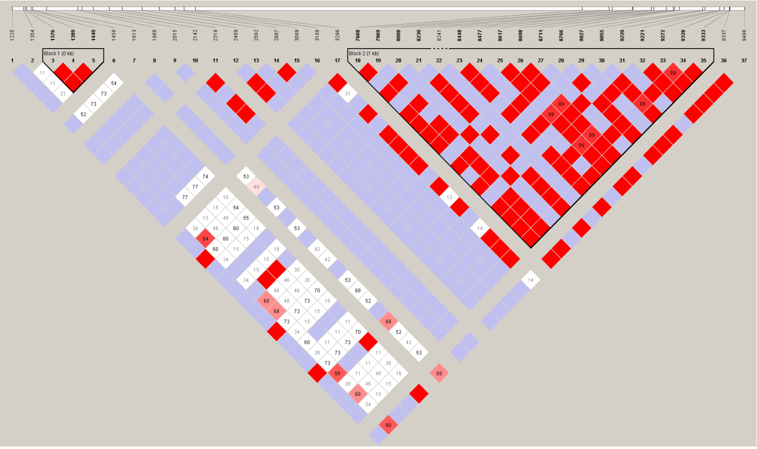

B.

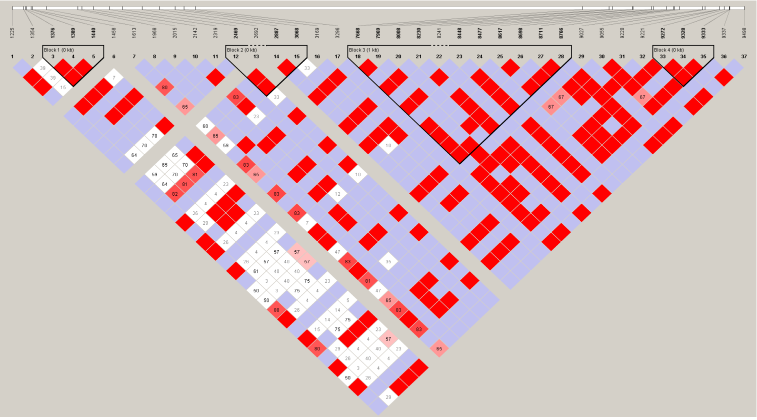

C.

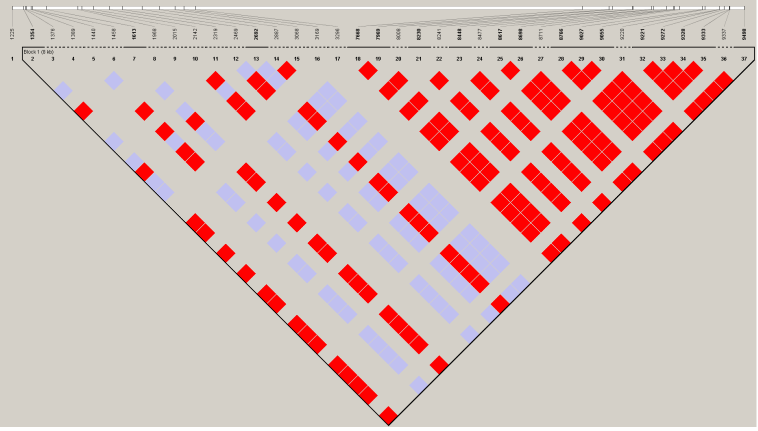

D.

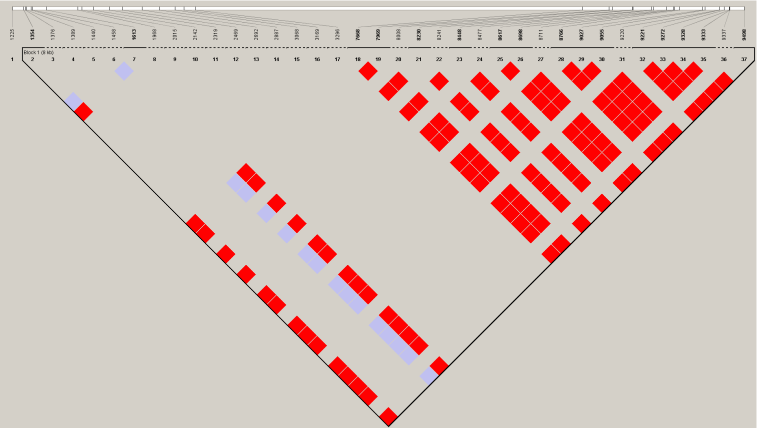

Additional File 2 Legend

Linkage disequilibrium analysis for the genomic region encompassing AVPR1B exons 1 and 2 for African American (A), Yorubans (B), European Americans (C) and Asians (D)
