# Supplementary material for: A complex selection signature at the human AVPR1B gene
Source: BMC Evol Biol. 2009 Jun 1;9:123. doi: 10.1186/1471-2148-9-123 (PMC2700802; doi:10.1186/1471-2148-9-123)
Supplement: Additional file 3 — Summary statistic p values obtained using different demographic models. [file 1471-2148-9-123-S3.pdf]

Additional File 3

Summary statistics *p* values for *AVPR1B* using different demographic models.

**Region**	**Pop**	**Voight *et al.* (2005)**			**Marth *et al.* (2004)**		
		D_T	D*	F*	D_T	D*	F*
Exon 1	**YRI**	.12	.33	.22	.077	.31	.18
	**AA**	.40	.12	.20	.31	.14	.23
	**EU**	.13	.053	.047	.21	.083	.074
	**AS**	.22	.22	.41	.43	.11	.18
Exon 2	**YRI**	.025	.024	.011	.013	.023	.0061
	**AA**	.019	.0034	.0033	.0095	.0009	.0015
	**EU**	0.11	.016	.029	.038	.0022	.0059
	**AS**	0.26	.023	.24	.49	.0013	.061

Marth GT, Czabarka E, Murvai J, Sherry ST: **The allele frequency spectrum in genome-wide human variation data reveals signals of differential demographic history in three large world populations.** *Genetics* 2004, **166**:351-372.

Voight BF, Adams AM, Frisse LA, Qian Y, Hudson RR, Di Rienzo A: **Interrogating multiple aspects of variation in a full resequencing data set to infer human population size changes.** *Proc Natl Acad Sci U S A* 2005, **102**:18508-18513.
